# Epoxides Derived from Dietary Dihomo-Gamma-Linolenic Acid Induce Germ Cell Death in *C. elegan*s

**DOI:** 10.1038/srep15417

**Published:** 2015-10-21

**Authors:** Marshall Deline, Julia Keller, Michael Rothe, Wolf-Hagen Schunck, Ralph Menzel, Jennifer L. Watts

**Affiliations:** 1 School of Molecular Biosciences and Center for Reproductive Biology, Washington State University, Pullman, WA 99614-6340, USA; 2 Humboldt-Universität zu Berlin, Department of Biology, Ecology, Philippstr. 13, House 18, 10115 Berlin, Germany; 3 Lipidomix GmbH, Robert-Rössle-Str. 10, 13125 Berlin, Germany; 4 Max Delbrück Center for Molecular Medicine, Robert-Rössle-Str. 10, 13125 Berlin, Germany

## Abstract

Dietary fats are not created equally, slight differences in structure lead to crucial differences in function. Muticellular organisms use polyunsaturated fatty acid as substrates to produce potent signaling molecules crucial for many physiological processes, including reproduction. Here we explored the mechanism responsible for germ cell loss induced by dietary supplementation of dihomo-gamma-linolenic acid (DGLA, 20:3n-6) in the roundworm *Caenorhabditis elegans*. In this study we found that *C. elegans* CYP-33E2 activity produces a range of epoxy and hydroxy metabolites from dietary DGLA. Knockdown of *cyp-33E2* suppressed the DGLA-induced sterility phenotype. Additionally, direct exposure of two specific DGLA-derived epoxy products, 8,9- and 14,15-epoxyeicosadienoic acids, produced germ cell abnormalities in the *C. elegans* gonad. We propose that sterility is mediated by the production of toxic DGLA-derived epoxides that trigger germ cell destruction. These studies are the first to establish a biological activity for a CYP-produced metabolite of DGLA.

Liberation of polyunsaturated fatty acids (PUFAs) from phospholipids leads to the formation of powerful, short range hormones influencing diverse functions necessary for development and physiology^1^,^2^. Cyclooxygenase and lipoxygenase enzymes act on PUFAs to produce prostaglandins, leukotrienes, and thromboxanes, collectively called eicosanoids^3^. The most well studied bioactive eicosanoids belong to series 2, indicating they are derived from arachidonic acid (AA, 20:4n-6). However, series 1 eicosanoids derived from dihomo-gamma-linolenic acid (DGLA, 20:3n-6) or series 3 eicosanoids derived from eicosapentaenoic acid (EPA, 20:5n-3) also exhibit biological activity. Eicosanoid mediators are best known for mediating pain and inflammation, but also regulate other physiological processes such as wound healing, bone metabolism, blood pressure, immune responses, ovulation, embryo implantation and the initiation of labor^4^,^5^,^6^. In Western diets, AA, an omega-6 fatty acid is often in excess, leading to an overproduction of pro-inflammatory eicosanoids. Dietary omega-3 fatty acids such as EPA, found in fish oil, are thought to be beneficial because eicosanoids derived from omega-3 fatty acids produce anti-inflammatory effects^7^.

A less understood class of eicosanoids are produced by cytochrome P450 monooxygenases (CYPs), which are a diverse collection of enzymes acting on various endogenous and xenobiotic molecules^8^. Fatty acid derived eicosanoids produced by the CYP pathway function in vascular dilation, angiogenesis, pain nociception and protection from ischemic/reperfusion damage^1^,^9^,^10^,^11^. In addition, epoxyeicosatrienoic acids (EETs) generated from AA by CYP epoxygenases were found to stimulate extensive multiorgan metastasis and escape from tumor dormancy in several tumor models^12^,^13^. Very few studies examining the direct effects of DGLA in mammals have been performed, although several cyclooxygenase- and lipoxygenase-derived eicosanoids appear to have anti-inflammatory and anti-proliferative properties^14^. Even though a great deal has been learned about eicosanoid formation and function, progress on elucidating beneficial and harmful functions of the thousands of diverse mediators has been limited due to the challenges imposed by low concentration, limited stability, and restricted knowledge of receptors for many eicosanoid species. In particular, the biosynthesis and biological activity of DGLA-derived CYP-eicosanoids has never been described before.

Studies using *C. elegans* are beginning to shed light on the activities of eicosanoids in developmental processes of invertebrates^2^,^15^. Our previous work has identified the involvement of PUFAs in the maintenance of germ cells. Specifically, wild type *C. elegans* become sterile in a dose-dependent manner when grown on plates containing DGLA^16^. This sterility phenotype is remarkably specific, as high concentrations of dietary omega-3 fatty acids with similar physical properties did not affect fertility. The sterility, caused by destruction of germ cells, is highly amenable to genetic manipulation. For example, the *fat-1* mutant strain, which produces excess endogenous DGLA^17^, or the *nhr-49* strain, which displays multiple lipid homeostasis defects^18^,^19^ become sterile at lower doses of DGLA than wild type. Conversely, strains with activated stress response pathways, such as *daf-2*, do not become sterile upon exposure to DGLA^20^. This suggests that fatty acid metabolism is highly regulated to protect against potentially toxic dietary metabolites.

When *C. elegans* are grown on elevated DGLA for their full life, their gonads consist of somatic cells which lack germ cells of any stage^16^. However, the impact of DGLA supplementation on germ cells can also be observed by short term DGLA supplementation of young adult worms with mature gonads. The *C. elegans* gonad consists of two U-shaped arms attached at one end by the uterus and spermatheca organs. A primordial germ cell niche is formed in the distal gonad where germ cells proliferate mitotically and transition into meiosis as they are pushed toward the proximal gonad to mature into sperm or oocytes^21^. Short term feeding of DGLA to young adult *C. elegans*, followed by visualization of the germ cell membrane and nuclei, indicated that DGLA supplementation leads first to the formation of multi-nucleated germ cells, then subsequently to germ cell apoptosis and cell clearance^20^. The rapid formation of multinucleated germ cells appears to be due to cell fusion of meiotic cells in the germline syncytium. Alternatively, failed cytokinesis during mitosis in the distal gonad could also account for the multinucleated cells.

In this study, we examined the mechanism in which dietary DGLA induces germ cell demise in *C. elegans*. Reverse genetic, biochemical, and morphological approaches were used to address the hypothesis that specific DGLA-derived eicosanoids have detrimental effects on germ cells survival. We found that CYP-33E2 activity produces hydroxy, epoxy, and diol products from dietary DGLA. However, only two of these metabolites, 8,9- and 14,15-epoxyeicosadienoic acid (EED), trigger the formation of multi-nucleated germ cells when injected directly into the *C. elegans* gonad syncytium. Thus, these oxygenated metabolites are likely responsible for sterility induced by dietary DGLA.

## Results

### Reduction of CYP-33E2 activity suppresses DGLA-induced sterility

**Figure 1:**
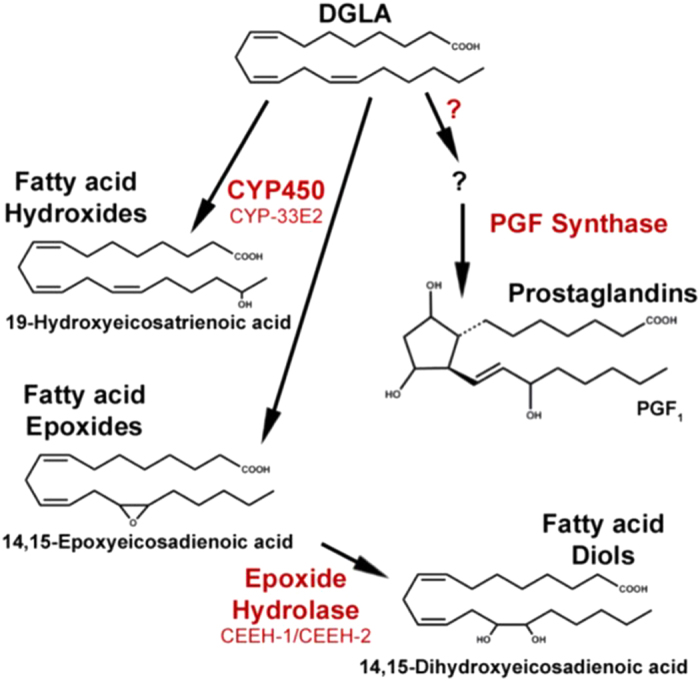
Schematic diagram of *C. elegans* DGLA cascade.

Physiological responses from PUFAs are often mediated by their conversion into oxygenated eicosanoid metabolites. To determine if DGLA-derived eicosanoids trigger the sterility induced by dietary DGLA, we used RNAi to inhibit the CYP and prostaglandin pathways that potentially metabolize DGLA (Fig. 1). In mammals, several CYP enzymes modify PUFAs, creating eicosanoids with epoxy (CYP2C and CYP2J isoforms) or hydroxy (CYP4A and CYP4F isoforms) modifications^8^.

**Figure 2:**
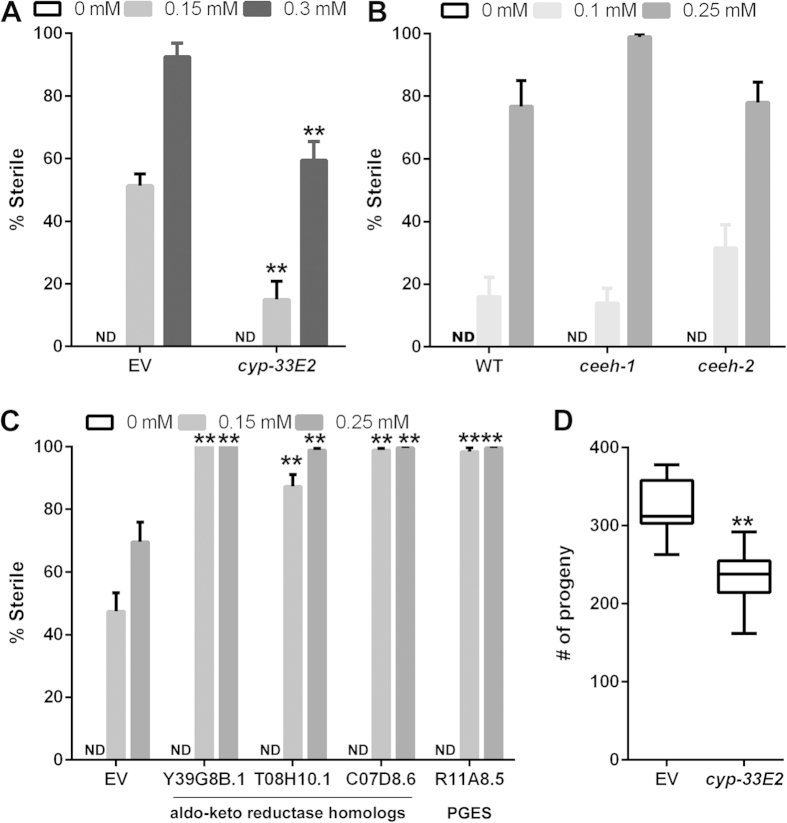
Genetic manipulation of DGLA cascade alters sterility from DGLA supplementation. Potential DGLA metabolism pathways were inhibited with RNAi or mutant alleles and assayed for sterility induced by DGLA supplementation. (**A**) RNAi knock-down of *cyp-33E2* suppresses DGLA-induced sterility. (**B**) *ceeh-1* and *ceeh-2* mutations do not significantly alter sterility. (**C**) Knockdown of alpha-keto reductase and PGE synthase homologs increases sensitivity to DGLA supplementation. (**D**) Knock-down of *cyp-33E2* decreases *C. elegans* reproduction success. For all panels, EV = empty vector control; ND = no detectable sterility on 0 mM DGLA; error bars, S.E.M, **P < 0.0005 compared to empty vector control.

The *C. elegans* homolog CYP-33E2 produces a range of hydroxy and epoxy metabolites from linoleic acid (18:2n-6), AA (20:4n-6) and EPA (20:5n-3)^22^. We reasoned that similar metabolites might be formed from dietary DGLA and might trigger germ cell demise, leading to sterility in *C. elegans*. If a specific oxygenated DGLA metabolite is toxic to germ cells, then knocking down the enzyme producing the metabolite would be predicted to suppress the sterility phenotype. We used RNAi to knock-down *cyp-33E2* and found, compared to empty vector controls, reduced populations of sterile worms when exposed to two concentrations of DGLA (Fig. 2A). GC/MS analysis revealed similar levels of DGLA uptake in the *cyp-33E2* RNAi (11.5% DGLA for 0.3 mM) as in the empty vector control (10.5% DGLA for 0.3 mM), revealing that the *cyp-33E2* is not simply blocking uptake or absorption of the dietary fatty acid. This result indicates that CYP-33E2 activity is involved in the process of germ cell destruction that leads to sterility in wild type worms supplemented with DGLA.

### Epoxide hydrolase and prostaglandin synthesis do not contribute to DGLA-induced sterility

Based on our previous studies^22^,^23^, we predicted that CYP-33E2 activity generates epoxy and hydroxy metabolites from DGLA. Because epoxides derived from fatty acids may be toxic^24^,^25^, intracellular concentrations of fatty acid epoxides are regulated by soluble epoxide hydrolase enzymes, which convert epoxides into diols. While this modification often aids in the clearance of epoxide molecules, in some cases the resulting diol is also toxic^24^,^25^. To determine if diol metabolites of DGLA may also be toxic and contribute to germ cell demise, we obtained a *ceeh-1* deletion and a *ceeh-2* mutant carrying an opal stop codon in exon 1 from the *C. elegans* “million mutant” project^26^, predicted to be a homolog of human soluble epoxide hydrolase^27^. The *ceeh-1* and *ceeh-2* mutants resulted in wild type levels of sterility on DGLA, though a slight but not significant increase in the number of sterile nematodes was seen in *ceeh-1* on 0.3 mM and in the *ceeh-2* mutant on 0.1 mM DGLA. (Fig. 2B). These experiments suggest that soluble epoxide hydrolase activity is not involved in DGLA-mediated toxicity.

In mammals, aldo-keto reductases (AKT) such as Prostaglandin F Synthase and isomerases such as Prostaglandin E Synthase act on prostaglandin H_1_ to produce series 1 F-class prostaglandins (PGF_1_)^4^. Although strong evidence exists for the synthesis of F-class prostaglandins in *C. elegans*^28^,^29^, the enzymes that catalyze the required cyclooxygenase activity have not been identified. We used RNAi knockdown of predicted homologs of human Prostaglandin E and F Synthases to determine if similar activities on DGLA were leading to the formation of a toxic metabolite. We tested three genes with ≥38% identity to human Prostaglandin F synthase: Y39G8B.1, T08H10.1, and C07D8.6 as well as R11A8.5 which has 45% identity to human Prostaglandin E Synthase 2. A significantly greater number of sterile nematodes were seen in the RNAi populations supplemented with DGLA than empty vector populations (Fig. 2C). This suggests instead that flux of DGLA through an alternative lipid metabolism pathway might prevent DGLA from conversion to more toxic eicosanoids produced by CYP-33E2.

### *C. elegans* CYP-33E2 activity promotes successful reproduction

To test whether CYP-33E2 impacts successful reproduction in untreated *C. elegans* wild type, the brood size of individual worms was measured. Inhibition of *cyp-33E2* resulted in a reduced brood size, approximately 30% lower than the empty vector control (Fig. 2D). Therefore, CYP-33E2 plays a positive role in reproduction under normal dietary conditions, possibly in the promotion of germ cell maintenance through eicosanoids synthesized from more abundant fatty acid substrates, such as eicosapentaenoic acid (EPA, 20:5n-3).

### *C. elegans* synthesize epoxy and hydroxy DGLA metabolites via CYP-33E2 activity

**Figure 3:**
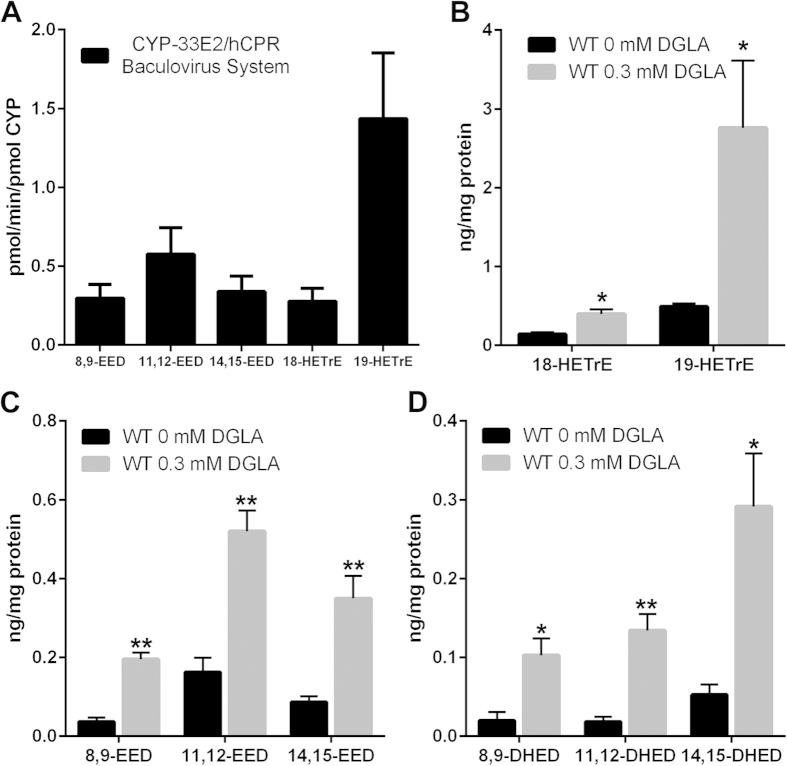
*C. elegans* DGLA metabolism is mediated by CYP activity. (**A**) Microsomes from a CYP-33E2/hCPR expressing baculovirus system were assayed with radiolabeled DGLA and separated by HPLC. The resulting products were identified and quantified by LC-MS/MS. (**B**–**D**) Endogenous DGLA metabolites of *C. elegans* fed 0 mM or 0.3 mM DGLA, assayed by LC-MS/MS. (**B**) Monohydroxy-metabolites (HETrE) increase in the presence of 0.3 mM DGLA. (**C**) Epoxy-metabolites (EED) and (**D**) Dihydroxy-metabolites (DHED) also increase in the presence of 0.3 mM DGLA. For all panels, error bars are S.E.M., *P < 0.05, **P < 0.001 compared to 0 mM DGLA.

Our genetic experiments infer that CYP-33E2 activity leads to toxic metabolite formation, while soluble epoxide hydrolases and conversion of DGLA into prostaglandins might protect worms from toxic DGLA-derived metabolites. To identify the DGLA metabolites produced by CYP-33E2 activity, microsomes from a baculovirus expression system co-expressing CYP-33E2 and human cytochrome P450 reductase (hCPR)^22^ were assayed with radiolabeled DGLA. Isolation and analysis of the resulting products by LC-MS/MS indicated that 8,9-, 11,12-, and 14,15-EEDs as well as the hydroxy derivatives, 18- and 19-hydroxyeicosatrienoic acids (HETrE) were produced by this monooxygenase system (Fig. 3A, Table 1). CYP-33E2 was previously shown to produce similar epoxy and hydroxy metabolites from EPA or AA substrates^22^. This indicates that epoxide and/or hydroxide containing DGLA metabolites may induce germ cell death.

To confirm that the DGLA metabolites produced by the recombinant CYP-33E2 are synthesized endogenously by *C. elegans*, the CYP-eicosanoid pattern from whole nematodes fed 0 mM or 0.3 mM DGLA were assayed by using LC-MS/MS. In addition to the epoxy and hydroxy metabolites found in the CYP-33E2 assay (Fig. 3B,C), 8,9-, 11,12-, and 14,15-di-hydroxyeicosadienoic acids (DHED), diols derived from the corresponding epoxides, were also produced (Fig. 3D). This suggests that endogenous epoxide hydrolase activity converts the corresponding epoxy metabolites to diols. Importantly, all three classes of eicosanoid metabolites were detected at a higher abundance in DGLA fed nematodes than in non-supplemented controls. This suggests that dietary supplementation of DGLA leads to the production of corresponding CYP-eicosanoids in the living worms.

### Functional assays of DGLA-derived eicosanoids

**Figure 4:**
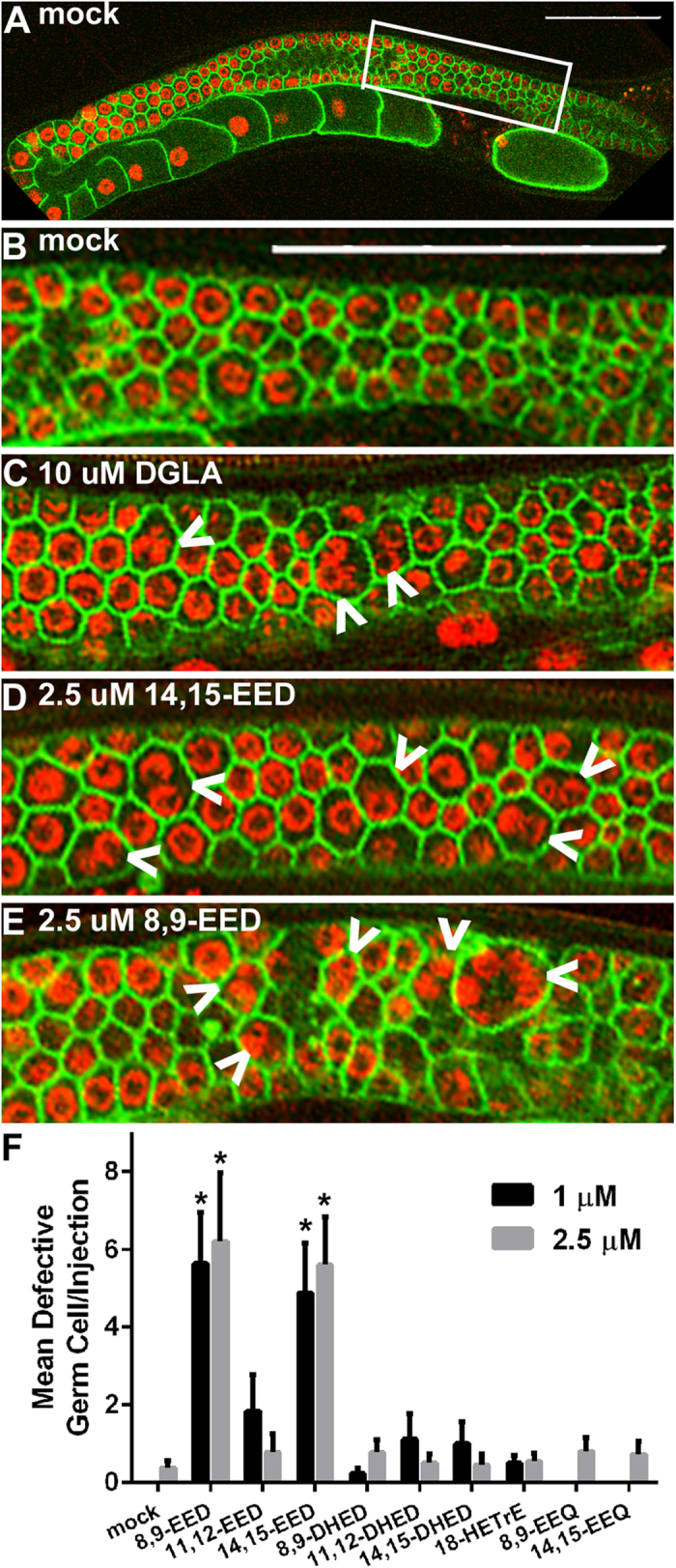
Specific epoxides derived from DGLA trigger germ cell abnormalities. Confocal microscopy of injected gonads indicates the defects produced by direct injection of specific DGLA metabolites. **(A**) Full gonad image of mock injection. White box indicates area of gonad enlarged for B–E. (**B**) Mock injected, (**C**) 10 μM DGLA injection, (**D**) 2.5 μM 14,15-EED, (**E**) 2.5 μM 8,9-EED. (**F**) The resulting defects are summarized as the mean number of defective germ cells per injection. White bars at the top right of A and B indicate 50 μm length. White arrows indicate multinucleated germ cells. Error bars, S.E.M, *P < 0.01 compared to mock injection. Injection of other DGLA metabolites did not result in increased germ cell defects compared to mock injections.

We sought evidence that specific DGLA metabolites induce the destruction of germ cells, leading to sterility. Of the DGLA metabolites found to be produced by *C. elegans*, only 14,15-EED was available commercially (Cayman Chemical). We produced the remaining metabolites by chemical oxidation (EEDs) following acidic hydrolysis (DHEDs) or by collection of CYP-33E2 produced 18-HETrE. All metabolites were purified by HPLC and quantified by MS. We developed a gonad injection assay in which the potency of specific metabolites could be directly assayed with smaller quantities of the purified eicosanoids. Because germ cells in the distal gonad are not completely enclosed, they share a common cytoplasm via a gonadal syncytium. Thus, multiple germ cells can be exposed to the metabolites from one injection. For proof of concept, DGLA was injected into the gonad of the *C. elegans* OD95 strain co-expressing fluorescent markers that target to the plasma membrane [GFP fusion that binds PI(4,5)P2] and chromosomes (mCherry-histone H2B)^30^. At a concentration of 10 μM, DGLA injections produced multinucleated, abnormal germ cells within several hours that were similar to those that developed when mature *C. elegans* were fed 0.3 mM DGLA (Fig. 4C). We then injected the purified DGLA-derived eicosanoids into gonads and observed gonad morphology 2–4 hours after injection. We found that injections of 8,9-EED or 14,15-EED at 1 and 2.5 μM produced multinucleated, abnormal germ cells in pre-meiotic and meiotic-transitioning germ cells (Fig. 4D–F). This suggests that these specific epoxide metabolites can trigger germ cell defects, because none of the other DGLA-derived eicosanoids produced the abnormal germ cell phenotype. Injections of DGLA at 1 and 2.5 μM did not produce abnormal germ cells compared to mock injections, suggesting that only a fraction of DGLA becomes modified into toxic metabolites, and therefore DGLA must be present at higher concentrations to manifest the effect. Our previous feeding studies showed that the sterility phenotype is highly specific to supplementation of DGLA. Similarly, we found that injection of the EPA metabolites 8,9- and 14,15-epoxyeicosatetraenoic acids (EEQs) did not cause the formation of abnormal germ cells. Thus, the germ cell toxicity is highly specific to the dietary DGLA-derived 8,9- and 14,15-EED eicosanoids.

## Discussion

Dietary DGLA causes *C. elegans* to become sterile due to destruction of germ cells during larval development. Through genetic, biochemical, and morphological studies, we demonstrate here that CYP-33E2 acts on dietary DGLA to produce two toxic metabolites, 8,9- and 14,15-EED, that mediate the destruction of germ cells. This is the first example of a DGLA-derived epoxide with physiological activity.

We showed that RNAi knockdown of CYP-33E2 produced nematodes that were resistant to DGLA-induced sterility, however, we found that RNAi targeting genes that potentially direct DGLA towards other lipid metabolism pathways increased the sensitivity of *C. elegans* towards DGLA. Prostaglandins are well known fatty acid metabolites involved in multiple aspects of reproduction. In mammals, prostaglandins functions include the induction of labor and oocyte maturation^31^. In *C. elegans,* F-class prostaglandins produced by PUFAs in oocytes mediate nematode sperm guidance towards oocytes^29^. Under favorable conditions with abundant nutrients, pheromones sensed by ciliated neurons stimulate prostaglandin synthesis in the ovary, which in turn attracts sperm to oocytes and promotes optimal reproduction^32^.

Even though CYP-33E2 activity mediates the toxic effects of dietary DGLA, it is likely that this enzyme produces beneficial PUFA metabolites that promote reproduction, because *cyp-33E2(RNAi)* nematodes produce a smaller brood than control when grown on normal (non-supplemented) *E. coli*. Previously, epoxy fatty acids had been shown to mostly act in vascular regulation and angiogenesis^1^. In mammals AA and EPA derived epoxide metabolites regulate blood pressure and renal function during pregnancy^33^. Additionally, the abundance of EETs in pre-ovulatory follicular cells follows the estrus cycle, and may influence estradiol formation^34^. In *C. elegans*, CYP-modified PUFA metabolites appear to regulate pharyngeal pumping, possibly through the activation of ion channels^22^,^23^. Recently, CYP-modified PUFAs, specifically 17,18- EEQ, was shown to impart protection against ischemic/reperfusion damage^9^,^35^. Thus, CYP-modified PUFA metabolites perform a range of important physiological functions in both vertebrates and invertebrates that are just beginning to be understood.

We previously showed that *C. elegans* mutant strains with constitutively active stress response pathways, including those resulting from inhibition of the insulin/IGF receptor DAF-2, were highly resistant to DGLA-induced sterility^20^. The *daf-2* mutant strain shows increased expression of phase-II detoxification genes, such as *gcs-1*, which encodes an enzyme required for glutathione biosynthesis^36^. Phase II detoxification enzymes could act to clear the DGLA epoxides before they incur damage to germ cells. On the other hand, our studies did not support the hypothesis that soluble epoxide hydrolases aid the clearance of the toxic epoxides, because the *ceeh-1* and *ceeh-2* mutants did not significantly enhance the sterility phenotype.

Based on our finding that knockdown of *cyp-33E2* leads to reduced brood size, we propose that under normal dietary conditions, polyunsaturated fatty acids are modified into epoxides by CYPs to promote successful reproduction. However, our studies demonstrate that an overabundance of DGLA in the diet leads to the synthesis of 8,9-EED and 14,15-EED, which may interfere with a physiological signal or act as toxins triggering germ defects and death. The specificity of these two isomers is critical to the germ cell phenotype, because exposure to 11,12-EED did not lead to germ cell abnormalities. Alternatively, our experiments do not rule out possibility that exposure to excess DGLA-derived epoxides lead to germ cell defects indirectly, for example, due to the disruption of signaling pathways that are regulated by a specific membrane lipid milieu^37^.

How the DGLA-derived epoxides induce germ cell abnormalities and germ cell death remains to be investigated. Similarly, the mechanisms of action for oxygenated fatty acids in mammalian processes such as vascular dilation are undefined. Though strong evidence indicates that epoxide fatty acid metabolites act in signal transduction, no receptors for these molecules have been identified^38^,^39^. Recently discovered functions for specific eicosanoids in *C. elegans*, such as the requirement for F-series prostaglandins in sperm guidance^28^,^29^,^32^, CYP involvement in response to hypoxia and re-oxygenation^9^,^35^, and the sensitivity of germ cells to oxygenated metabolites of dietary DGLA, demonstrate the utility of the genetically amenable *C. elegans* system for the discovery and elucidation of cellular mechanisms of eicosanoid signaling in physiological processes.

## Methods

### *C. elegans* maintenance and strains

Strains used in this study include: Wild type (N2 Bristol), RB2321 [*ceeh-1(ok3153)*], VC20710 [*ceeh-2(gk379719)*], and OD95 [Itls37, Itls38]^30^. The VC20710 strain was outcrossed 5x to N2. Nematode growth media (NGM) was used to maintain *C. elegans* with *E. coli* strain OP50 at 20 °C. Feeding RNAi was performed on NGM plates supplemented with 100 μg/ml ampicillin and 2 mM isopropyl-β-D-thiogalactopyranoside using the Ahringer lab RNAi library clones in *E. coli* strain HT115^40^,^41^. All RNAi constructs were sequence verified using Sanger sequencing.

### Sterility assay

Wild-type strains were supplemented with DGLA on either standard NGM or RNAi medium as previously described^16^,^20^,^42^. Light microscopy was used to identify sterile worms as a lack of embryos in the adult uterus. For each concentration of DGLA, the percentage of sterile worms in the population was determined by scoring at least 30 worms per plate as fertile or sterile, and averaging the sterility of populations on 4–8 plates. Comparisons between sterility levels of control populations and experimental populations were made using two-tailed student’s t tests. Exogenous DGLA uptake was confirmed by gas chromatography/mass spectrometry (GC/MS) of nematodes^16^,^20^,^42^.

### CYP-33E2 activity on DGLA

*C. elegans* CYP-33E2 and hCPR were co-expressed in Sf9 cells as previously described^22^. For the enzymatic activity assay, microsomes with 9 pmol of CYP-33E2 were incubated with 10 μM [1-^14^C]-DGLA (ARC, St. Louis, MO, USA) at 25 °C for 10 minutes. The analysis of the obtained metabolites were performed by RP-HPLC and LC-MS/MS were prepared as previously described^22^.

### Endogenous CYP-produced DGLA metabolites

Endogenous eicosanoids of *C. elegans* were identified exactly as previously described^22^. The synthesized DGLA metabolites were identified by their molecular weight and their characteristic fragmentation pattern under collision induced dissociation. The mass of negative molecular ions are m/z = 321 for epoxides and hydroxides and m/z = 339 for diols. The characteristic cleavage beside the oxygen functional group allows the differentiation of all regioisomeres (Table 1). The retention time was in an expected range compared to a wide spectrum of well-known oxylipins.

### Production of DGLA derived eicosanoids

Whereas only 14,15-EED was available commercially (Cayman Chemical), the both other regioisomeric monoepoxides were synthesized by reacting the parental DGLA, supplemented with trace amounts of the corresponding [1-^14^C]-labeled compound, with m-chloroperoxybenzoic acid as described previously for the chemical oxidation of AA to EETs^43^. To produce EED derived diols (DHEDs) we hydrolyzed each of the three epoxides with 1 mL of a 1:1 acetic acid/water mixture over night at 45 °C while shaking with 70 rpm. All reaction products were resolved and purified by RP- and subsequent NP-HPLC as described in detail previously^44^. 18- and 19-HETrE were produced enzymatically by recombinant CYP-33E2/hCPR activity as described above and isolated after RP-HPLC purification. The identity and quantity of all regioisomeric metabolites was confirmed by LC-MS/MS.

### Fatty acid and eicosanoid injection assay

For proof of concept, sodium dihomo-gamma-linolenic acid was dissolved in water at a concentration of 10 μM. All other fatty acids and metabolites arrived in ethanol and were diluted to 1 or 2.5 μM with water, mock injections used 1% ethanol. A glass capillary tube (World Precision Instruments Inc., TW100F-4) was drawn into a needle, loaded, and used to inject into the gonad syncytium of the OD95 strain using an inverted light microscope. For each concentration of fatty acid metabolite, 8–12 individual nematodes were injected. Nematodes were recovered on NGM seeded with *E. coli* for 2 hours at 20˚C. Nematodes were then transferred to a drop of 0.1 mg/mL levamisole on a 2% agarose pad and a coverslip was applied. Germ cell membrane and nuclei were viewed with laser excitation at 488 and 561 nm (filtered at 495/535 nm and 600/670 nm for GFP and mCherry excitation/emission) and imaged on a Leica TCS SP5 confocal microscope with a 63X oil immersion lens. Images were processed identically using Adobe Photoshop CS4. The mean number of defective germ cells per injected worm was determined, and two-tailed student’s t test was used to compare the number of defective germ cells in injected worms compared to mock injections.

## Additional Information

**How to cite this article**: Deline, M. *et al.* Epoxides Derived from Dietary Dihomo-Gamma-Linolenic Acid Induce Germ Cell Death in *C. elegans*. *Sci. Rep.* **5**, 15417; doi: 10.1038/srep15417 (2015).

## Figures and Tables

**Table 1:** LC-MS/MS parameters obtained from the analysis of DGLA-derived CYP-eicosanoids.

	Precursor Ion	ProductIon I	ProductIon II
18-HETrE	321	263	303
19-HETrE	321	277	303
8,9-EED	321	181	169
11,12-EED	321	163	157
14,15-EED	321	221	209
8,9-DHED	339	227	169
11,12-DHED	339	187	157
14,15-DHED	339	209	129

## References

[b1] Spector A. A. & Kim H. Y. Cytochrome P epoxygenase pathway of polyunsaturated fatty acid metabolism. Biochim Biophys Acta 1851, 356–365 (2015). 25093613 10.1016/j.bbalip.2014.07.020 PMC4314516

[b2] Vrablik T. L. & Watts J. L. Polyunsaturated fatty acid derived signaling in reproduction and development: insights from Caenorhabditis elegans and Drosophila melanogaster. Mol Reprod Dev 80, 244–259 (2013). 23440886 10.1002/mrd.22167 PMC4350910

[b3] Astarita G., Kendall A. C., Dennis E. A. & Nicolaou A. Targeted lipidomic strategies for oxygenated metabolites of polyunsaturated fatty acids. Biochim Biophys Acta 1851, 456–468 (2015). 25486530 10.1016/j.bbalip.2014.11.012 PMC4323855

[b4] Funk C. D. Prostaglandins and leukotrienes: advances in eicosanoid biology. Science 294, 1871–1875 (2001). 11729303 10.1126/science.294.5548.1871

[b5] von Moltke J. *et al.* Rapid induction of inflammatory lipid mediators by the inflammasome *in vivo*. Nature 490, 107–111 (2012). 22902502 10.1038/nature11351 PMC3465483

[b6] Brock T. G. Regulating leukotriene synthesis: the role of nuclear 5-lipoxygenase. J Cell Biochem 96, 1203–1211 (2005). 16215982 10.1002/jcb.20662

[b7] Smyth E. M., Grosser T., Wang M., Yu Y. & FitzGerald G. A. Prostanoids in health and disease. J Lipid Res 50 Suppl, S423–428 (2009). 19095631 10.1194/jlr.R800094-JLR200 PMC2674745

[b8] Nebert D. W., Wikvall K. & Miller W. L. Human cytochromes P450 in health and disease. Philos Trans R Soc Lond B Biol Sci 368, 20120431 (2013). 23297354 10.1098/rstb.2012.0431 PMC3538421

[b9] Ma D. K. *et al.* Cytochrome P450 drives a HIF-regulated behavioral response to reoxygenation by C. elegans. Science 341, 554–558 (2013). 23811225 10.1126/science.1235753 PMC3969381

[b10] Schuck R. N. *et al.* The cytochrome P450 epoxygenase pathway regulates the hepatic inflammatory response in fatty liver disease. PLoS One 9, e110162 (2014). 25310404 10.1371/journal.pone.0110162 PMC4195706

[b11] Wagner K., Vito S., Inceoglu B. & Hammock B. D. The role of long chain fatty acids and their epoxide metabolites in nociceptive signaling. Prostaglandins Other Lipid Mediat 113–115, 2–12 (2014). 10.1016/j.prostaglandins.2014.09.001 PMC4254344 25240260

[b12] Wang D. & Dubois R. N. Epoxyeicosatrienoic acids: a double-edged sword in cardiovascular diseases and cancer. J Clin Invest 122, 19–22 (2012). 22182836 10.1172/JCI61453 PMC3248310

[b13] Panigrahy D. *et al.* Epoxyeicosanoids stimulate multiorgan metastasis and tumor dormancy escape in mice. J Clin Invest 122, 178–191 (2012). 22182838 10.1172/JCI58128 PMC3248288

[b14] Wang X., Lin H. & Gu Y. Multiple roles of dihomo-gamma-linolenic acid against proliferation diseases. Lipids Health Dis 11, 25 (2012). 22333072 10.1186/1476-511X-11-25 PMC3295719

[b15] Zhu H. & Han M. Exploring developmental and physiological functions of fatty acid and lipid variants through worm and fly genetics. Annu Rev Genet 48, 119–148 (2014). 25195508 10.1146/annurev-genet-041814-095928

[b16] Watts J. L. & Browse J. Dietary manipulation implicates lipid signaling in the regulation of germ cell maintenance in C. elegans. Dev Biol 292, 381–392 (2006). 16487504 10.1016/j.ydbio.2006.01.013 PMC1584401

[b17] Watts J. L. & Browse J. Genetic dissection of polyunsaturated fatty acid synthesis in Caenorhabditis elegans. Proc Natl Acad Sci USA 99, 5854–5859 (2002). 11972048 10.1073/pnas.092064799 PMC122866

[b18] Van Gilst M. R., Hadjivassiliou H., Jolly A. & Yamamoto K. R. Nuclear hormone receptor NHR-49 controls fat consumption and fatty acid composition in C. elegans. PLoS Biol 3, e53 (2005). 15719061 10.1371/journal.pbio.0030053 PMC547972

[b19] Van Gilst M. R., Hadjivassiliou H. & Yamamoto K. R. A Caenorhabditis elegans nutrient response system partially dependent on nuclear receptor NHR-49. Proc Natl Acad Sci USA 102, 13496–13501 (2005). 16157872 10.1073/pnas.0506234102 PMC1201344

[b20] Webster C. M., Deline M. L. & Watts J. L. Stress response pathways protect germ cells from omega-6 polyunsaturated fatty acid-mediated toxicity in Caenorhabditis elegans. Dev Biol 373, 14–25 (2013). 23064027 10.1016/j.ydbio.2012.10.002 PMC3508147

[b21] Hubbard E. J. & Greenstein D. Introduction to the germ line. (September 1, 2005), *WormBook*, ed. The C. elegans Research Community, WormBook, doi/10.1895/wormbook. 1. 18. 1 http://www.wormbook.org. 10.1895/wormbook.1.18.1 PMC4781435 18050415

[b22] Kosel M. *et al.* Eicosanoid formation by a cytochrome P450 isoform expressed in the pharynx of Caenorhabditis elegans. Biochem J 435, 689–700 (2011). 21309752 10.1042/BJ20101942

[b23] Kulas J., Schmidt C., Rothe M., Schunck W. H. & Menzel R. Cytochrome P450-dependent metabolism of eicosapentaenoic acid in the nematode Caenorhabditis elegans. Arch Biochem Biophys 472, 65–75 (2008). 18282462 10.1016/j.abb.2008.02.002

[b24] Kosaka K., Suzuki K., Hayakawa M., Sugiyama S. & Ozawa T. Leukotoxin, a linoleate epoxide: its implication in the late death of patients with extensive burns. Mol Cell Biochem 139, 141–148 (1994). 7862104 10.1007/BF01081737

[b25] Zheng J., Plopper C. G., Lakritz J., Storms D. H. & Hammock B. D. Leukotoxin-diol: a putative toxic mediator involved in acute respiratory distress syndrome. Am J Respir Cell Mol Biol 25, 434–438 (2001). 11694448 10.1165/ajrcmb.25.4.4104

[b26] Thompson O. *et al.* The million mutation project: a new approach to genetics in Caenorhabditis elegans. Genome Res 23, 1749–1762 (2013). 23800452 10.1101/gr.157651.113 PMC3787271

[b27] Harris T. R. *et al.* Identification of two epoxide hydrolases in Caenorhabditis elegans that metabolize mammalian lipid signaling molecules. Arch Biochem Biophys 472, 139–149 (2008). 18267101 10.1016/j.abb.2008.01.016 PMC2435305

[b28] Edmonds J. W. *et al.* Insulin/FOXO signaling regulates ovarian prostaglandins critical for reproduction. Dev Cell 19, 858–871 (2010). 21145501 10.1016/j.devcel.2010.11.005 PMC3026445

[b29] Hoang H. D., Prasain J. K., Dorand D. & Miller M. A. A heterogeneous mixture of F-series prostaglandins promotes sperm guidance in the Caenorhabditis elegans reproductive tract. PLoS Genet 9, e1003271 (2013). 23382703 10.1371/journal.pgen.1003271 PMC3561059

[b30] McNally K., Audhya A., Oegema K. & McNally F. J. Katanin controls mitotic and meiotic spindle length. J Cell Biol 175, 881–891 (2006). 17178907 10.1083/jcb.200608117 PMC2064698

[b31] Nunes C., Silva J. V., Silva V., Torgal I. & Fardilha M. Signalling pathways involved in oocyte growth, acquisition of competence and activation. Hum Fertil (Camb) 18, 149–55 (2015). 25738216 10.3109/14647273.2015.1006692

[b32] McKnight K. *et al.* Neurosensory perception of environmental cues modulates sperm motility critical for fertilization. Science 344, 754–757 (2014). 24833393 10.1126/science.1250598 PMC4094289

[b33] Huang H. *et al.* Epoxyeicosatrienoic Acid inhibition alters renal hemodynamics during pregnancy. Exp Biol Med (Maywood) 231, 1744–1752 (2006). 17138762 10.1177/153537020623101112

[b34] Newman J. W. *et al.* Cytochrome p450-dependent lipid metabolism in preovulatory follicles. Endocrinology 145, 5097–5105 (2004). 15308618 10.1210/en.2004-0710

[b35] Keller J. *et al.* CYP-13A12 of the nematode Caenorhabditis elegans is a PUFA-epoxygenase involved in behavioural response to reoxygenation. Biochem J 464, 61–71 (2014). 25138176 10.1042/BJ20140848

[b36] An J. H. & Blackwell T. K. SKN-1 links C. elegans mesendodermal specification to a conserved oxidative stress response. Genes & development 17, 1882–1893 (2003). 12869585 10.1101/gad.1107803 PMC196237

[b37] Kniazeva M., Shen H., Euler T., Wang C. & Han M. Regulation of maternal phospholipid composition and IP(3)-dependent embryonic membrane dynamics by a specific fatty acid metabolic event in C. elegans. Genes & development 26, 554–566 (2012). 22426533 10.1101/gad.187054.112 PMC3315117

[b38] Ding Y. *et al.* The biological actions of 11,12-epoxyeicosatrienoic acid in endothelial cells are specific to the R/S-enantiomer and require the G(s) protein. J Pharmacol Exp Ther 350, 14–21 (2014). 24763066 10.1124/jpet.114.214254 PMC4062412

[b39] Akbulut T. *et al.* 20-HETE activates the Raf/MEK/ERK pathway in renal epithelial cells through an EGFR- and c-Src-dependent mechanism. Am J Physiol Renal Physiol 297, F662–670 (2009). 19570883 10.1152/ajprenal.00146.2009 PMC2739708

[b40] Kamath R. S. & Ahringer J. Genome-wide RNAi screening in Caenorhabditis elegans. Methods 30, 313–321 (2003). 12828945 10.1016/s1046-2023(03)00050-1

[b41] Kamath R. S., Martinez-Campos M., Zipperlen P., Fraser A. G. & Ahringer J. Effectiveness of specific RNA-mediated interference through ingested double-stranded RNA in Caenorhabditis elegans. Genome Biol 2, RESEARCH0002 (2001). 11178279 10.1186/gb-2000-2-1-research0002 PMC17598

[b42] Deline M. L., Vrablik T. L. & Watts J. L. Dietary supplementation of polyunsaturated fatty acids in Caenorhabditis elegans. J Vis Exp 81, 10.3791/50879 (2013). PMC3992124 24326396

[b43] Falck J. R., Yadagiri P. & Capdevila J. Synthesis of epoxyeicosatrienoic acids and heteroatom analogs. Methods Enzymol 187, 357–364 (1990). 2233353 10.1016/0076-6879(90)87042-2

[b44] Arnold C. *et al.* Arachidonic acid-metabolizing cytochrome P450 enzymes are targets of {omega}-3 fatty acids. J Biol Chem 285, 32720–32733 (2010). 20732876 10.1074/jbc.M110.118406 PMC2963419

